# Polyherbal formulation conjugated to gold nanoparticles induced ferroptosis in drug-resistant breast cancer stem cells through ferritin degradation

**DOI:** 10.3389/fphar.2023.1134758

**Published:** 2023-03-27

**Authors:** Prasanthi Chittineedi, Arifullah Mohammed, Mohammad Khairul Azhar Abdul Razab, Norazlina Mat Nawi, Santhi Latha Pandrangi

**Affiliations:** ^1^ Onco-Stem Cell Research Laboratory, Department of Biochemistry and Bioinformatics, School of Science, GITAM (Deemed to be) University, Visakhapatnam, India; ^2^ Department of Agriculture Science, Faculty of Agro-Based Industry, Universiti Malaysia Kelantan, Jeli, Kelantan, Malaysia; ^3^ Medical Radiation Programme, School of Health Sciences, Universiti Sains Malaysia, Health campus, Kubang Kerian, Kelantan, Malaysia; ^4^ Department of Nuclear Medicine, Radiotherapy and Oncology, School of Medical Sciences, Universiti Sains Malaysia, Health campus, Kubang Kerian, Kelantan, Malaysia

**Keywords:** polyherbal formulation, gold nanoparticles, drug-resistance, cancer stem cells, ferroptosis

## Abstract

**Aim:** Due to their minimal side effects, the anti-cancer properties of the polyherbal formulation are being investigated. However, due to their low absorption potential, the administration of polyherbal formulations is restricted. Loading the polyherbal formulation into gold nanoparticles enhances the bioavailability of the polyherbal formulation (PHF) accompanied by reducing the concentration of doxorubicin (dox). Ferroptosis is one of the novel pathways that specifically target cancer stem cells due to high ferritin levels. Hence, in the present study, we conjugated polyherbal formulation with gold nanoparticles and studied its effect on inducing ferroptosis in drug-resistant breast cancer cell lines.

**Materials and methods:** PHF and dox conjugated to gold nanoparticles were characterized using FTIR, UV-Vis spectrophotometer, DLS, particle size analyzer, and XRD. The drug entrapment and efficiency studies were performed to assess the biodegradable potential of the synthesized gold nanoparticles. Paclitaxel-resistant breast cancer stem cells were generated, and an MTT assay was performed to evaluate the cytotoxicity potential of AuNP-PHF and AuNP-dox. Scratch assay and clonogenic assay were performed to assess the migration and proliferation of the cells after treatment with chosen drug combinations. The ability of PHF and dox conjugated to gold nanoparticles to induce ferritinophagy was evaluated by RT-PCR. Finally, image analysis was performed to check apoptosis and cellular ROS using inverted fluorescent microscope. The ability to induce cell cycle arrest was assessed by cell cycle analysis using flow cytometer.

**Results and conclusion:** PHF and dox conjugated to gold nanoparticles showed high stability and showed to induce ferritin degradation in drug resistant breast cancer stem cells through ferritin degradation. AuNP-PHF in combination with low dose of AuNP-Dox nanoconjugate could be used as an effective cancer therapeutic agent, by targeting the autophagy necroptosis axis.

## 1 Introduction

Breast Cancer stands in first place in overall incidence of disease particularly in females. Although conventional therapies were practiced to treat breast cancer, cancer relapse/recurrence pose a great threat to eradicate the tumor cells completely lseading to tumor-free microenvironment (Pandrangi et al., 2014a). Cancer stem cells (CSCs) regarded as tumor-initiating cells by Bonnet and Disk are responsible for the increased rate of tumor relapse/recurrence (Hermann et al., 2010). These cells show the genotypic characteristics of normal stem cells (NSCs) are highly proliferative and are resistant to conventional therapies. Additionally, resistance to chemotherapeutic drugs is one of the major concerns that is interlinked with patients’ prognosis and survival rates.

Resistance to chemotherapeutic drugs is accompanied with apoptotic resistance because the chemotherapeutic drugs are majorly focused on inducing apoptosis (Li et al., 2021). Hence, novel pathways that could trigger tumor cell and CSC death is of utmost important. Ferritinophagy is one such novel pathway which is independent of caspase activation and is dependent on iron degradation through autophagy (Gao et al., 2016). Mounting evidences suggest that both cancer and CSCs accumulate bulk iron reserves in the form of ferritin to sustain the vital tumor microenvironment (Recalcati et al., 2019). On the other hand, literature demonstrates that serum ferritin levels serve as prognostic factor to predict the incidence of tumor relapse/recurrence (Chittineedi et al., 2022a). Hence, iron metabolism in cancer and CSCs is highly altered (Pandrangi et al., 2014b; Basuli et al., 2017; Chen et al., 2019; Recalcati et al., 2019; Wang et al., 2019). Additionally, recent research on the role of ferritin in these cells revealed that ferritin plays a double-edged sword role in cancer and CSCs by regulating both proliferation and death (Pandrangi et al., 2022a). However, the mechanism through which ferritin is being targeted plays a crucial role.

Studies by Chanvarachote et al. (2016) revealed that iron induced in CSCs and aggressive phenotypes of human lung cancer cell lines. Through enhance of the expression of SOX9 which is an important regulator of stemness and an important marker for tracheal differentiation and its formation (Chanvorachote and Luanpitpong, 2022). Conversely, when ferritin was degraded in drug-resistant cervical CSCs by pharmacological inhibitors, resulted in elevated ROS which is the hallmark of tumor cell death (Chittineedi et al., 2022b).

Due to their less toxicity and very low to negligible side-effects therapeutic properties of natural, active compounds of plants were being investigated. Polyherbal formulation (PHF) which comprises mixture of active compounds show to have promising therapeutic effects against various diseases including cancer (Atanasov et al., 2015; Latha Pandrangi et al., 2022). However, biodegradation, poor bioavailability due to hydrophobicity are the major limitations of these bioactive compounds. However, these limitations could be overcome by loading these drugs into vehicles, such as nanoparticles. Gold nanoparticles (AuNPs) gained much attention due to its less toxicity (Rajesh, 2000; Biazar et al., 2011; Wicki et al., 2015; Zheng et al., 2020; Rambatla et al., 2021). Hence, in the present study we hypothesized that loading PHF and Doxorubicin (dox) into AuNPs could yield better therapeutic efficacy when compared with unloaded drugs. The purpose of choosing dox is to reduce the concentration of it without altering its therapeutic potential. This is because, literature suggests that although dox is one of the potential anti neoplastic drugs, it showed enhanced risk of cardiotoxicity. Hence, in the present study we focused on synthesizing AuNPs to load PHF and dox to the nanoparticles and assess the potential of ferritinophagy induction in paclitaxel-resistant breast cancer stem cells (PacR/MCF-7CSCs).

## 2 Materials and methods

### 2.1 Extraction and preparation of aqueous polyherbal formulation (PHF) and doxorubicin

PHF containing herbs Rauwolfia serpentina, Garcinia indica, and Terminalia arjuna were used in the present study and was procured from Vaidhya Narayana Murthy Cancer Medicine, Karnataka, India. The powder was packed in filter paper and extracted using methanol in the Soxhlet apparatus. The Soxhlet was run till the colourless solution had been obtained. Once the colourless solution had been obtained, the solution was collected and dried using rotary evaporator. The dried crude extract was dissolved in UltraPure DNase/RNase-free distilled water (Gibco) to obtain a 1 mg/mL concentration. The extract was filtered using a 0.2 µ syringe filter and stored at −20°C till further analysis.

Dox was commercially purchased from Sigma and a concentration of 5 mg/mL was prepared using 1xPBS and filtered using a 0.2 µ syringe filter. The obtained solution was stored at 4°C till further analysis.

### 2.2 Binding of the desired drugs with gold nanoparticles

Gold nanoparticles were commercially purchased from HiMedia (MBNPG001) and diluted to 8 μg/mL. Two combinations of AuNP-drug were made. Briefly, for first combination 3 parts of 8 μg/mL of AuNP were mixed with 1 part of 1 mg/mL plant extract and 1.2 μg/mL of doxorubicin (dox) separately (1:3 ratio). For second combination, 5 parts of 8 μg/mL of AuNP were mixed with 1 part of 1 mg/mL plant extract and 1.2 μg/mL of doxorubicin (dox) separately (1:5 ratio). The mixture was placed on the magnetic stirrer and stirred overnight at room temperature. The next day, the mixture was subjected to ultracentrifugation at 13,000 rpm for 10 min, and the supernatant was separated from the pellet. The pellet was dissolved in nuclease-free water.

### 2.3 Drug entrapment/encapsulation efficiency (EE)

Drug entrapment efficiency was calculated to assess the percentage of drug encapsulated into the gold nanoparticles. Briefly, the OD of both supernatants obtained from the ultracentrifugation and the bare plant extract and dox was measured at 490 nm, 520 nm, and 595 nm. The encapsulation efficiency was calculated using the formula:

$$EE\;\%=\left(OD\;of\;drug-OD\;of\;untraped\;drug\;in\;supernatant\right)/\left(OD\;of\;added\;drug\right)*100$$

### 2.4 UV visible spectroscopy

Absorption spectra of the synthesized AuNPs and AuNPs encapsulated with PHF and dox nanocomposites were monitored using a double-beam UV spectrophotometer (*Shimadzu UV-1800*). The prepared AuNP-drugs were diluted to 3 mL using 1xPBS, transferred to 1 cm UV-quartz cell, and the absorption spectra were recorded.

### 2.5 Fourier transform infraRed spectroscopy (FTIR)

FTIR measurements were carried out using an FT-IR spectrometer (Brucker) in the range of 500–4500 cm^−1^. 10 µL of each AuNP-PHF and AuNP-Dox were placed on the probe, and the values were recorded.

### 2.6 Zeta potential and particle size analysis

Surface charge which represents zeta potential and particle size of the nanoparticles play a crucial role in determining the stability and potential to penetrate into the nucleus respectively. Hence, to determine the surface charge and particle size, both AuNP-PHF and AuNP-Dox nanocomposites were analyzed through Horiba SZ-100 based on the principle of photon correlation spectroscopy. The average zeta potential was determined using 60 s as the analysis time.

### 2.7 Powder X-ray diffraction

The crystal structures of the conjugated AuNP-PHF and AuNP-Dox X-ray Diffractometer were carried out using Brucker D8 advance XRD using copper cell. Briefly, the liquid samples were casted on to the glass slide (1.5*1.5 cm), air dried and 2 theta values were measured in XRD equipped with Cu anode filter and D8 diffractometer at setting of 40kV/30 mA to recognize the crystalline status of the nanocomposites.

### 2.8 Cell line maintenance

MCF-7 cell line was procured from NCCS, Pune, and was cultured in Dulbecco’s Modified Eagle Media (DMEM, Invitrogen, Carlsbad, CA, United States) comprising 10% FBS (Gibco), 1% Antibiotics (Invitrogen, Carlsbad, CA, United States), 1% Glutamax, and 5 μg/mL insulin (Sigma-Aldrich, St. Louis, MO, United States). The cells were grown in 5% CO_2_ incubator till the flask was 90% confluent, expanded by trypsinization and were used for *in vitro* analysis.

### 2.9 Development of drug resistant clones

Breast cancer (MCF-7) cells were exposed to increasing concentration of Paclitaxel for 48 h and cell survival was assessed by an MTT assay to study the kinetics of cell death. Briefly, the cells were replenished with fresh media containing varying concentrations of Paclitaxel and incubated at 37°C for 48 h in 5% CO_2_-saturated atmosphere. Cells grown in drug-free media were chosen as control. After incubation, the cells were washed and replenished with 200 μL of fresh media, 50 μL of MTT solution (Sigma-Aldrich, St. Louis, MO, United States) were added, and further incubated for 3 h followed by addition of 200 μL of DMSO to dissolve the formed formazan crystals. The absorbance was measured immediately at 570 nm using a multi-well spectrophotometer (BIO-RAD PR4100). The absorbance of control cells was taken as 100% viable cells and the values of treated cells were calculated as a percentage of the controls. The obtained IC50 value was used to generate drug-resistant MCF-7 clones. To develop acquired drug-resistant cells, breast cancer cell lines are exposed to increasing concentrations of paclitaxcel. Briefly, MCF-7 cells that were 80% confluent were exposed to the IC50 of paclitaxel (30 nM) and incubated for another 48 h. Subsequently, the drug-containing medium was withdrawn, fresh media was added and the cells were allowed to grow. After reaching 80% confluency, the cells were again trypsinized and were re-exposed to double the dose of the above drugs. This process was repeated at least 10 times (10 cycles) and the MTT assay is performed on these developed drug-resistant clones.

### 2.10 Cytotoxicity assay

To evaluate the IC50 of the characterized PHF, an MTT assay was performed. Briefly, an increasing concentration of PHF, AuNP-PHF, AuNP-Dox drugs were added to the 96-well plate containing PacR/MCF-7cells and was incubated for 24 h. After incubation, the cells were fed with 200 μL of fresh medium, 50 μL of MTT (Sigma-Aldrich, St. Louis, MO, United States) solution was added, and the plates were incubated for 3 h. After incubation, MTT-containing media was withdrawn, the formed formazan crystals were dissolved by adding 200 μL of DMSO and the absorbance was measured at 570 nm using a multi-well spectrophotometer (BIO-RAD PR4100).

### 2.11 Migration assay

The cell migration potential of PacR/MCF-7cell lines was analyzed by performing wound healing assay. Briefly, the cell lines were seeded in 35 mm Petri plates and incubated at 37°C in a CO_2_ incubator until the cells were 90% confluent. The wells were then scratched using a scrapper across the centre of the plate, and the desired combination of drugs (PHF, AuNP-PHF + AuNP-Dox, and Dox) were added after subsequent washes. Untreated cells were taken as control. The wound healing capacity was monitored for every 12 h and the images were recorded.

### 2.12 Clonogenic assay

To determine the formation of colonies by the cancer cells treated with the appropriate concentration of hibiscus plant extract, the cells trypsinized cells were seeded with a density of 1 × 10^3^ cells in individual 35 mm Petri plates. The cells were incubated with desired combination of drugs (PHF, AuNP-PHF + AuNP-Dox, and Dox) were added. Untreated cells were chosen as control. After incubation, the fresh media was added by withdrawing drug containing media and the images of colonies were captured using an inverted microscope.

### 2.13 Gene expression analysis

To study the effect of AuNP-loaded PHF in inducing ferritinophagy RT-PCR analysis was performed. Briefly, PacR/MCF-7cells were treated with the drug concentrations equivalent to the IC50 of PHF, IC50 of Dox, combination of IC50 of AuNP-PHF and IC25 of AuNP-Dox and incubated for 24 h. Untreated cells were used as control. After incubation, RNA was isolated using TriZol and immediately converted into cDNA using a cDNA master mix (Abcam). For analysing the expression of various ferritinophagy markers such as LC-IIIB, NCOA4, Ftn, primers specific to these genes were added to the cDNA along with SYBR green. The reaction mixture was then placed in a thermal cycler (Applied Biosystems) and RT-PCR was carried out taking GAPDH as an internal control. Sequences of the primers used were listed in Table 1.

**TABLE 1:** List of primers used in the present study.

Gene	Forward primer	Reverse primer
Ferritin	GCT​CTA​CGC​CTC​CTA​CGT​TT	GTG​GCC​AGT​TTG​TGC​AGT​TC
LC-3B	CAG​CGT​CTC​CAC​ACC​AAT​CT	GCG​GGT​TTT​GTG​AAC​CTG​AA
NCOA4	GGG​CAA​CCT​CAG​CCA​GTT​AT	GGG​ATC​TGA​AAA​TTC​CCA​ACG​G
Ferritin	GCT​CTA​CGC​CTC​CTA​CGT​TT	GTG​GCC​AGT​TTG​TGC​AGT​TC
GPX-4	ATTGGTCGGCTGGACGAG	CCG​AAC​TGG​TTA​CAC​GGG​AA
GAPDH	ACA​GTC​AGC​CGC​ATC​TTC​TT	GGC​AAC​AAT​ATC​CAC​TTT​ACC

### 2.14 Cell cycle analysis

To determine at which stage the cell has been inhibited to undergo cell division, we performed a cell cycle analysis using a flow cytometer. PacR/MCF-7cells treated with desired drug combinations for 24 h, along with untreated cells. Cells were collected and pelleted down. The pellet was then washed twice with 1X PBS. After completing the washing steps, the pellet is suspended in 50 µL of 100 μg/mL RNase and 200 µL of 50 μg/mL Propidium Iodide and immediately analyzed cells using a flow cytometer (BD Acquri).

### 2.15 AO/EtBr dual apoptosis staining

To visualise apoptotic cells, acridine orange/ethidium bromide (AO/EtBr) staining was performed. Briefly, both drug treated and untreated cells were initially stained with 100 μg/mL acridine orange and incubated at dark for 10 min. After incubation, the cells were washed with 1XPBS and stained with 100 μg/mL ethidium bromide for 10 min. To avoid background noise cells were washed with 1XPBS and the images of apoptotic cells were captured in OLYMPUS inverted fluorescent microscope.

### 2.16 Cellular ROS assay

For visualizing cellular ROS, drug-treated and control cells were stained with 20 µM DCF-DA (Sigma-Aldrich) for 30 min at dark. Excess stain was removed by PBS wash and the cells for counterstained with Hoechst 33342 for 5 min at dark to visualise cellular nuclei. Cells were washed with PBS to avoid background noise and were visualised in EVOS FLc inverted fluorescent microscope (Invitrogen).

### 2.17 Statistical analysis

All the experiments were done in triplicates and the data is presented as mean values. Student *t*-test is performed to compare the difference. Pearson co-efficient was done to measure the significance for the generated spheroids. *p*-value <0.05 was considered to be statistically significant.

## 3 Results

### 3.1 Drug entrapment efficiency

**FIGURE 1:**
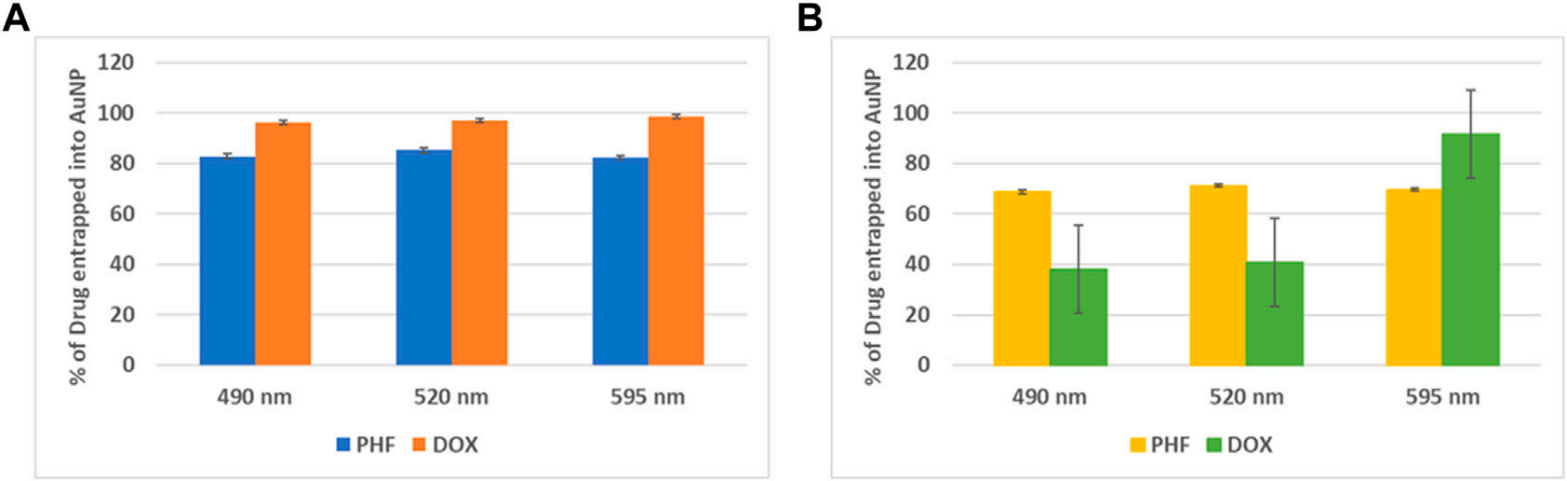
Graphical Representation of maximum entrapment efficiency of AuNP-PHF and AuNP-Dox. To quantify the amount of drug loaded into AuNPs, absorption at different wavelengths was measured. **(A)** depicts the % of drug entrapped into AuNP at 1:3 dilution. While **(B)** depicts the % of drug entrapped into AuNP at 1:5 dilution. As shown in the above figure, 1:3 dilution gave best results with an entrapment efficiency of 85.2% with maximum absorbance at 520 nm for AuNP-PHF and an entrapment efficiency of 98.47% with maximum absorbance at 595 nm for AuNP-dox. (*p* < 0.05).

To calculate the concentration of PHF and Dox that has been encapsulated into the gold nanoparticles, the drugs and AuNPs in a ratio of 1:3 and 1:5 were stirred continuously for about 24 h, ultracentrifuged at 10,000 rpm for 15 min, and the absorbance at 490 nm, 520 nm, and 595 nm of the supernatant was recorded in a plate reader. The entrapment efficiency of the AuNP-PHF and AuNP-Dox were calculated using the formula (Section 2.2). As shown in Figure 1, the obtained values suggest that the drug was efficiently entrapped at 1:3 ratio with maximum absorbance at 520 nm for AuNP-PHF and 595 nm for AuNP-dox with 85.2% and 98.47% entrapment efficiency respectively.

### 3.2 UV-vis spectrophotometer

**FIGURE 2:**
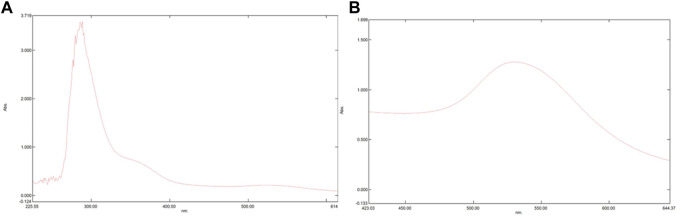
Pictorial representation of peaks formed in UV-Vis Spectrophotometer. UV-Vis Spectrophotometer is used to assess the maximum absorption of a particular compounds thereby predicting surface plasmon resonance. The shift in peak demonstrates that there is a change in the chemical composition of a particular compound. As shown in the above figure, there is shift from 280 nm to 300 nm in **(A)** which represents the spectrophotometric analysis of AuNP-PHF, while in **(B)** which depicts the spectrophotometric analysis of AuNP-Dox with a peak shift from 280 nm to 530 nm. Since AuNPs were commercially purchased with maximum absorption at 280 nm analysis for AuNPs was not performed.

To confirm the conjugation of PHF and Dox to AuNPs UV-visible spectroscopy was performed. AuNPs procured from HiMedia with a maximum absorption at λ_max_ ∼530 nm were was used in the current study. As depicted in Figure 2 the maximum absorption of UV-vis light of AuNP-PHF and AuNP-Dox were considerably shifted from λ_max_ ∼530 nm to λ_max_ ∼300 nm and λ_max_ ∼580 nm respectively, confirming that both PHF and Dox probably conjugated onto the surfaces of the AuNPs, suggesting that their photo-physical properties were modified. The position of the maximum absorption peak and its width depends on numerous factors, such as the dielectric environment, aggregation of the particles, morphology of the particles, and sometimes due to the 2^0^ metabolites that are responsible for the synthesis which is regarded as surface plasmon resonance (SPR).

### 3.3 FTIR

**FIGURE 3:**
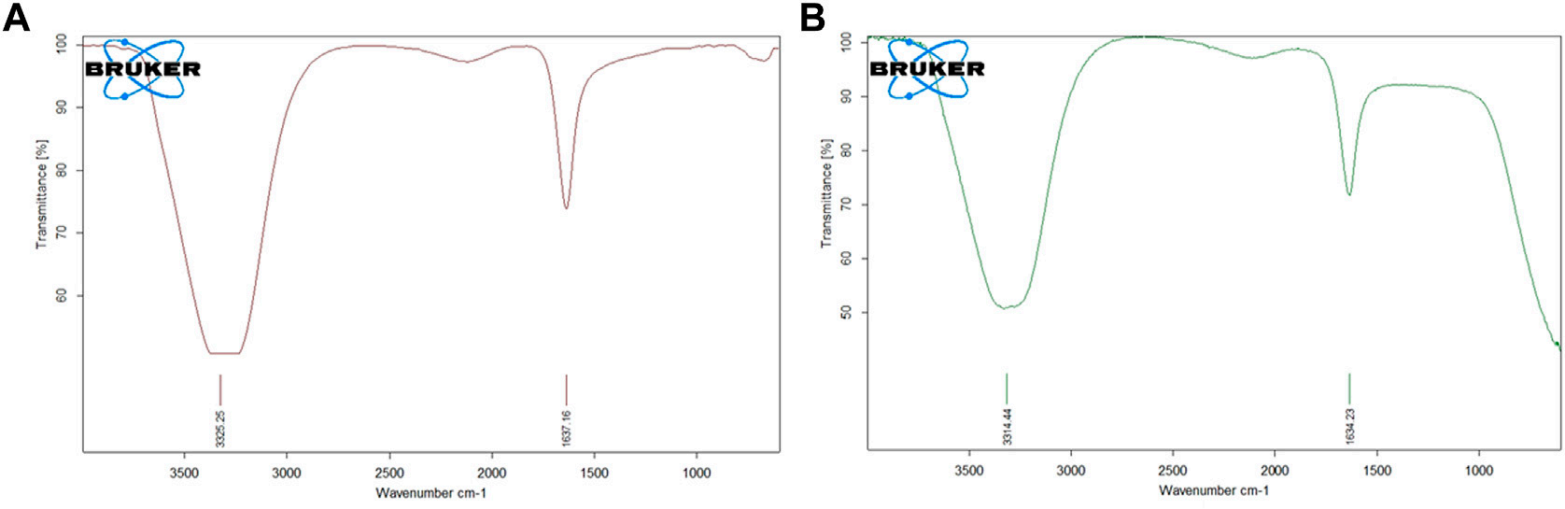
Pictorial representation of FTIR analysis of AuNP-PHF and AuNP-Dox. FTIR analysis is used to predict whether the drug molecules were loaded into AuNPs. As shown in the above figure, both the drug combinations were effectively loaded in the nanoparticles. The obtained results suggest that the amide and phenol groups of the drugs formed a layer to the AuNP, thereby serving as a capping agent protecting AuNP from agglomeration leading to enhanced stability of the AuNPs. **(A)** FTIR analysis of AuNP-PHF; **(B)** FTIR analysis of AuNP-Dox.

FTIR analysis is crucial for the identification of the functional groups that are present in the AuNP-PHF and AuNP-Dox. The FTIR spectrum of AuNP-PHF and AuNP-Dox were recorded in the spectral region 3000–300 cm^−1^ and were depicted in Figure 3. The FTIR spectrum of AuNPs conjugated with PHF and Dox showed the O-H symmetric stretching vibration frequency of phenols at 3325 cm^−1^ for AuNP-PHF and 3315 cm^−1^ for AuNP-dox, signifying the presence of alcohol. Additionally, the presence of the N-H bond of primary amines was recorded at 1637 cm^−1^ for AuNP-PHF and 1634 cm^−1^ for AuNP-dox, suggesting the presence of an amide group. The obtained results demonstrate that the amide and phenol groups of the drugs formed a layer on AuNP, thereby serving as a capping agent protecting AuNP from agglomeration leading to enhanced stability of AuNPs.

### 3.4 Analysis of zeta potential and particle size

**FIGURE 4:**
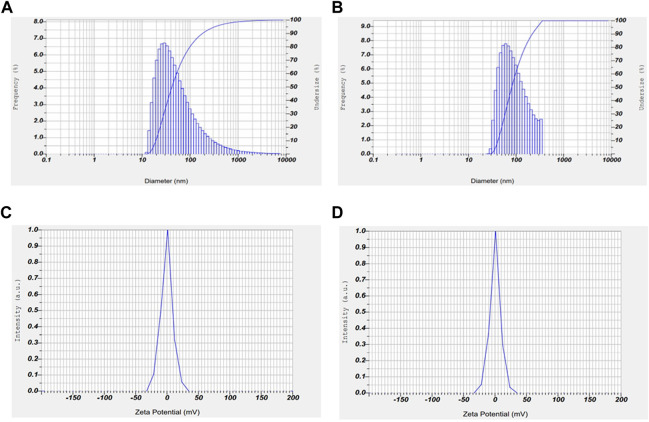
Graphical representation of zeta potential and particle size of AuNP-PHF and AuNP-Dox. To check the stability and particle size of the drug loaded nanoparticles, Zeta potential and particle size analysis was carried out using DLS. As shown in the above figure, the obtained results suggest that AuNP-PHF and AuNP-Dox showed 35.1 nm particle size and −0.6 mV zeta potential; 1.9 nm particle size and 0.2 mV zeta potential, respectively suggesting that the drug loaded nanoparticles are highly stable and capable of penetrating into the nucleus. **(A)** represents the particle size of AuNP-dox; **(B)** represents the particle size of AuNP-PHF; **(C)** represents the Zeta potential of AuNP-PHF; **(D)** represents the Zeta potential of AuNP-dox.

Zeta potential and particle size are two important characteristics of nanoparticles which determines the stability and potential to penetrate into the nucleus respectively. The stability and size distribution of the prepared nanoparticles were determined using zeta sizer. The average particle size and zeta potential of AuNP-PHF and AuNP-Dox were measured to be 35.1 nm and −0.6 mV; 1.9 nm and 0.2 mV, respectively. The encapsulated PHF and dox have shown high surface stability (Figure 4).

### 3.5 XRD

**FIGURE 5:**
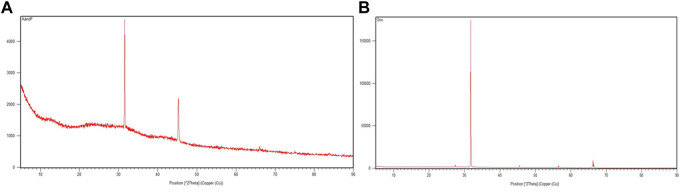
X-ray Diffraction peaks of AuNP-PHF and AuNP-Dox. To check the crystalline state of the conjugated drugs, XRD analysis was performed and 2 theta values were obtained. **(A)** represents the XRD peaks of AuNP-PHF; **(B)** represents the XRD peaks of AuNP-Dox.

The crystal structures of AuNP-PHF and AuNP-Dox were examined by XRD (Figure 5). AuNP-PHF showed two intense peaks at 28.2^0^ and 31.5^0^. While AuNP-Dox showed one intense peak at 31.7^0^ and 7 short peaks at 27.4^0^, 28.5^0^, 45.4^0^, 56.5^0^, 56.6^0^, 66.2^0^, and 66.4^0^ respectively, confirming the polycrystalline face-centered cubic structure of the synthesized AuNP-PHF and AuNP-Dox.

### 3.6 Development of paclitaxel-resistant breast cancer cells

To generate PacR/MCF-7cells, breast cancer cells were exposed to increasing concentrations of paclitaxel for about 10 cycles, and cellular cytotoxicity was measured using an MTT assay after each cycle. Interestingly, we observed that after the 6th cycle, there was no further increase in the IC50 of MCF-7 cell lines, suggesting that the MCF-7 cell lines acquired drug resistance to paclitaxel with an IC50 of 90 nM, indicating that the drug-resistant cells showed three times more IC50 than that of MCF-7 cells.

### 3.7 Determination of IC50 value through MTT assay

MTT assay was performed on PacR/MCF-7 cell lines using PHF, AuNP-PHF, AuNP-Dox, and Dox to determine the IC50 in the cells. The number of viable cells gradually decreased with an increase in the concentration of the drug. The IC50 value was determined from the standard graph, which was plotted with a concentration of MTT reagent against absorbance measured at 570 nm. The obtained IC50 absorbance was used to calculate the concentration of the drug. Obtained results suggest that PacR/MCF-7 cells showed an IC50 of 50 μg/mL for PHF, 20 μg/mL for AuNP-PHF, 1.9 μg/mL for AuNP-Dox, and for 3.7 μg/mL Dox respectively suggesting that encapsulation of PHF and Dox into AuNPs reduced the IC50 values of the respective drugs.

### 3.8 Migration assay

**FIGURE 6:**
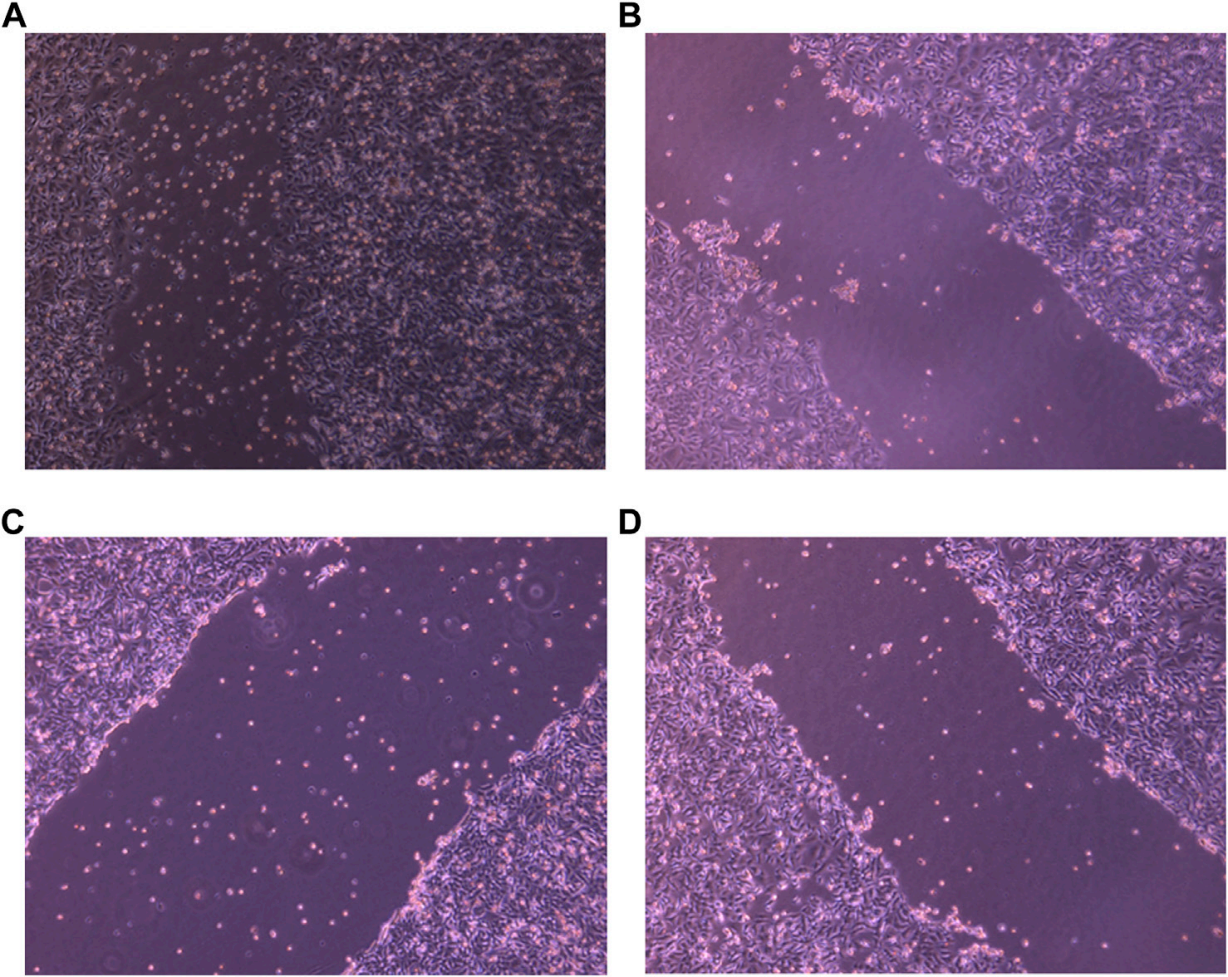
Representative pictures of wound healing ability of PacR/MCF-7 cell lines. Briefly, a wound was created using a scrapper in 35 mm Petri plate with 90%–100% confluent cells. The cells were then exposed to PHF (**(B)**), AuNP-PHF in combination with AuNP-Dox (6 **(C)**), and Dox (6 **(D)**) for 24 h. Untreated cells were chosen as control (6 **(A)**). As depicted in the figure, the scratch was almost closed in control cells while a consistent breadth of scratch was observed in drug-treated cells suggesting that the chosen drugs potentially inhibited the metastasis ability of PacR/MCF-7 cells.

The ability of a tumor cell to migrate and invade other tissues denotes its aggressive nature and migration potential. A potent anti-tumor drug is supposed to inhibit the migration ability, leading to reduction in tumor metastasis. To check this, a scratch was made using a scrapper and these cells were exposed to desired drug concentrations and specific drug combinations. Untreated cells were chosen as negative control. After exposing them to the drugs, the breadth of the scratch was examined under the microscope. As shown in the Figures 6, PHF, AuNP-PHF in combination with AuNP-Dox and Dox could not be able close the wound even after 36 h when compared to the wound of control cells which was almost healed by 36 h suggesting that the chosen drug combinations potentially inhibited the metastatic ability of PacR/MCF-7.

### 3.9 Clonogenic assay

**FIGURE 7:**
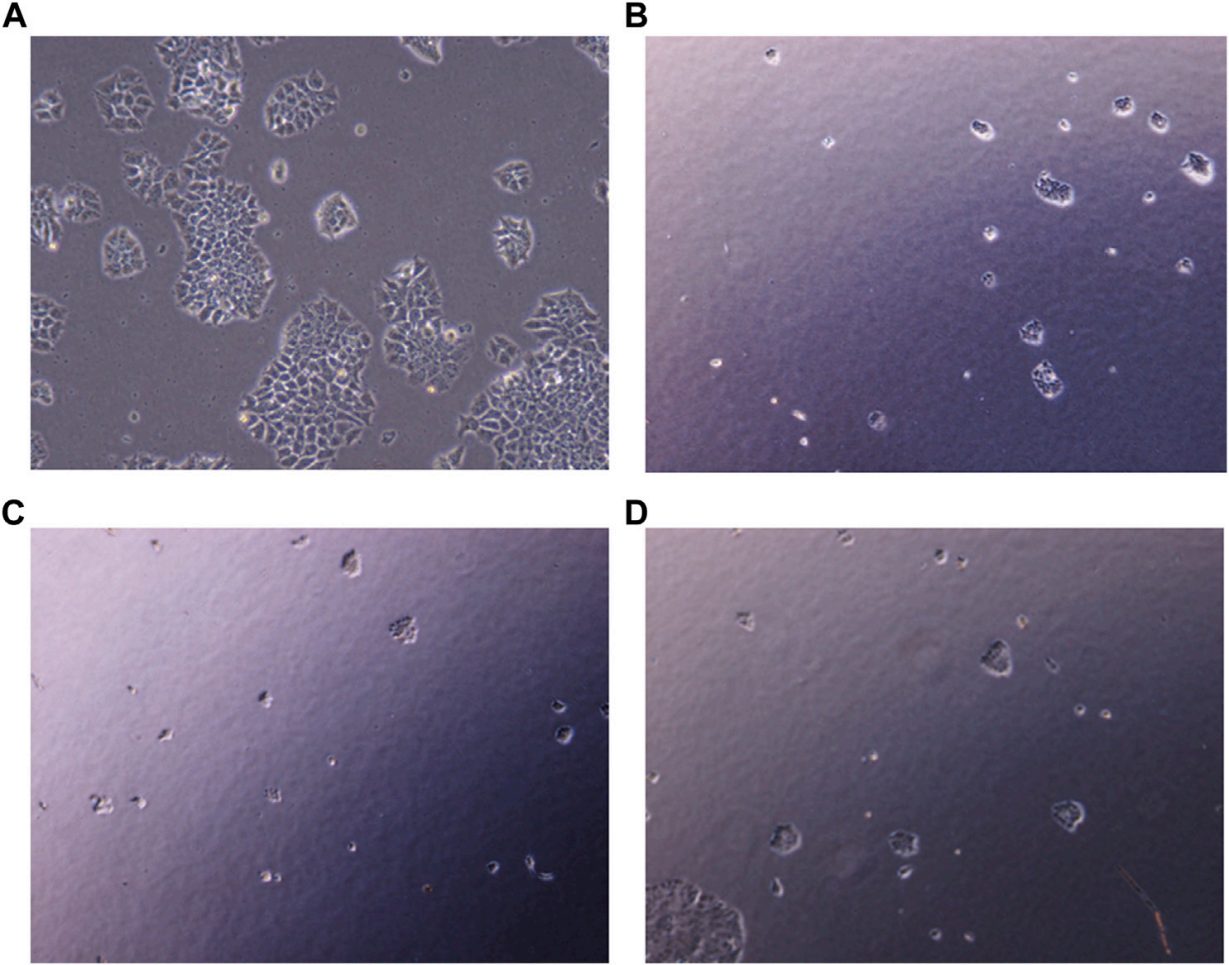
Representative pictures of clonogenic ability of PacR/MCF-7 cell lines. Briefly, single cells were plated on 35 mm Petri plates and incubated for 24 h. Once the cells were adhered, they were exposed to PHF (7 **(B)**), AuNP-PHF in combination with AuNP-Dox (7 **(C)**), and Dox (7 **(D)**) for 24 h. Untreated cells were chosen as control (7 **(A)**). After incubation, the cells were visualised and the number of colonies formed were recorded. As depicted in the figure, all the drug combinations potentially halted colony formation thereby reduced the tumor-initiating capability.

To explore the effect of PHF in combination with low concentration of Dox and AuNP-PHF in combination with AuNP-Dox on colony formation, single cells were plated on Petri plates, and desired drug treatment was given. Untreated cells were chosen as negative control, while Dox was chosen as positive control. After drug treatment, the cells were checked for their ability to form colonies. As expected, both PHF and Dox alone and AuNP-PHF in combination with AuNP-Dox potentially attenuated the colony formation. Figure 7 shows the total number of colonies obtained in controls and drug-treated PacR/MCF-7 cells.

### 3.10 Gene expression analysis using RT-PCR

**FIGURE 8:**
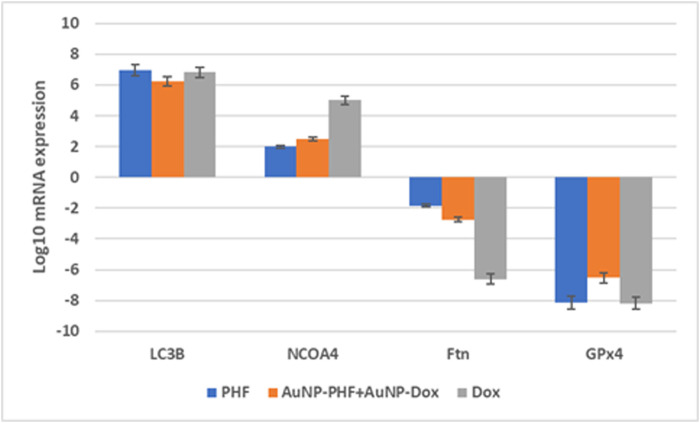
Gene expression profile of ferritinophagy markers obtained after RT-PCR analysis. Real time PCR amplifies the gene present in the sample with the help of the picked primers specific to the gene of interest. Obtained data suggests that the ferroptinophagic markers with oncogenic activity were downregulated. While ferroptinophagic markers serving as tumor suppressors have been significantly upregulated. (*p* < 0.05).

After exposing PacR/MCF-7to the chosen drug combinations (IC50 AuNP-PHF, IC25 AuNP-Dox, IC50 PHF, and IC25 Dox, IC50 Dox), RT-PCR was carried out to analyze the expression of ferritinophagy markers. RT-PCR analysis suggested that the negative regulators of ferritinophagy, i.e., ferritin and GPx4, were downregulated while NCOA4 and LCIIIB, regarded as positive modulators of ferritinophagy, have been upregulated (Figure 8).

### 3.11 Cell cycle analysis

**FIGURE 9:**
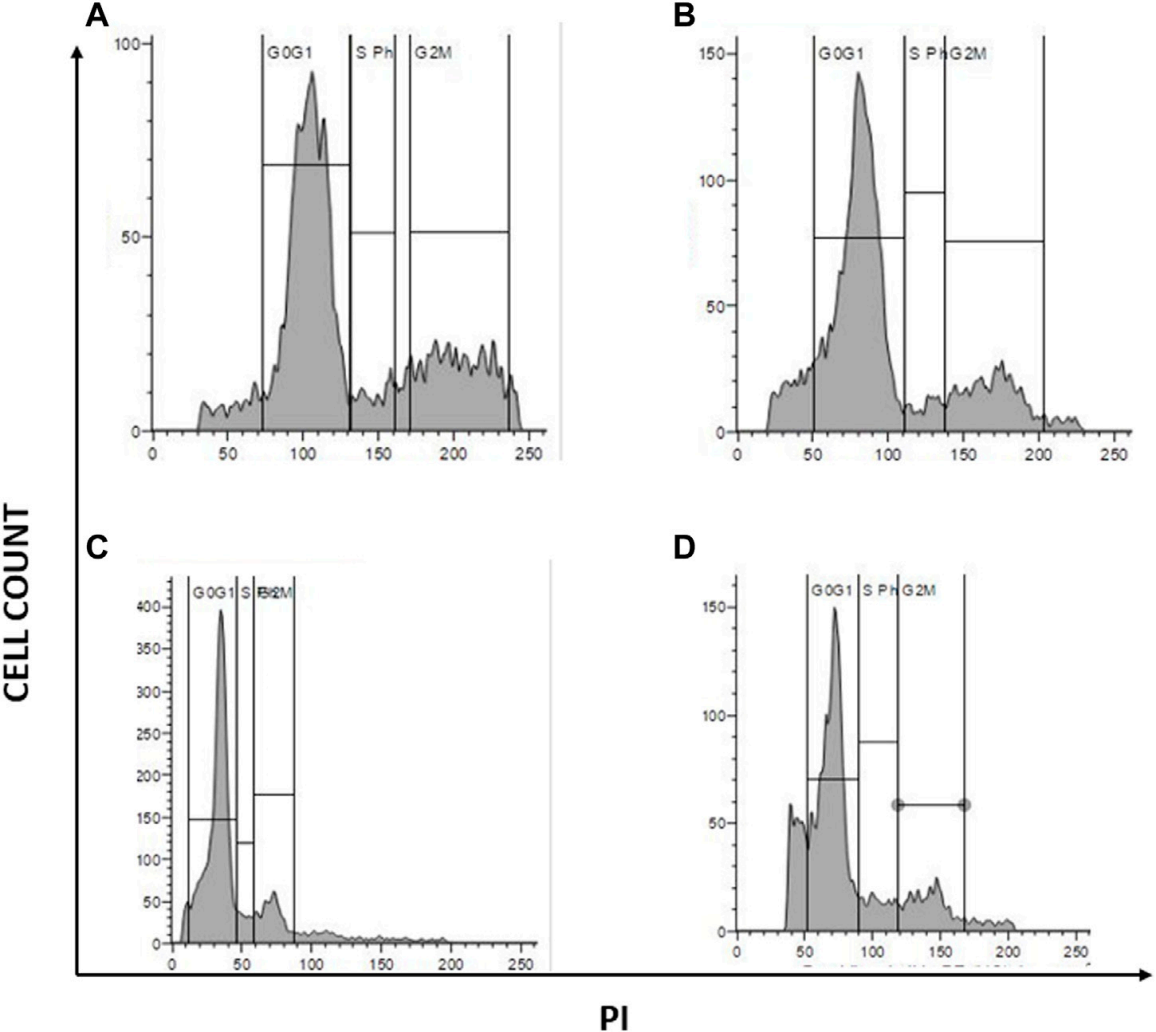
Graphical representation of cell cycle analysis acquired through flow cytometer. represents the quantification of cell cycle analysis. **(A)** Represents PacR/MCF-7CSCs control; **(B)** represents PacR/MCF-7CSCs treated with IC50 of PHF alone; **(C)** represents PacR/MCF-7CSCs treated with combination of IC50 of AuNP-PHF and IC25 of AuNP-dox; **(D)** represents PacR/MCF-7CSCs treated with IC50 of dox alone. As shown in the Figure 7, there is a rise in percentage of cells from 58.53% in control to 66.2% and 68.75 in the G0/G1 phase in PacR/MCF-7cells treated with IC50 PHF and combination of IC50 of AuNP-PHF and IC25 of AuNP-dox respectively, suggesting that these drugs induced G0/G1 arrest. While dox alone induced S-phase arrest with an increase in the percent of cells in S-phase from 6.12 in control to 8.84 in dox-treated PacR/MCF-7 cells.

Due to the elevated expression of various growth factors, tumor cells exhibit irregular cell proliferation. The ability of a compound to arrest cell cycle progression at specific checkpoints leading to sensitization of tumor cells to cell death is regarded as a hallmark of novel antineoplastic drugs. To further investigate the effects of the combination of AuNP-polyherbal formulation along with AuNP-dox to induce ferritinophagy, their effect on cell progression has been checked. Briefly, PacR/MCF-7 cells were stained with PI, and the progression of cells into various phases of the cell cycle was monitored. The obtained data revealed that the cells had been accumulated in the G0/G1 phase of the cell cycle in both the groups (AuNP-PHF in combination with AuNP-dox and PHF with dox) with an increase from 58.53% in control to 66.2% and 68.75 in the G0/G1 phase respectively in PacR/MCF-7 cells. While dox alone induced S-phase arrest with an increase in the percent of cells from 6.12 in control to 8.84 in dox-treated PacR/MCF-7cells (Figure 9).

### 3.12 Dual AO/EtBr staining

**FIGURE 10:**
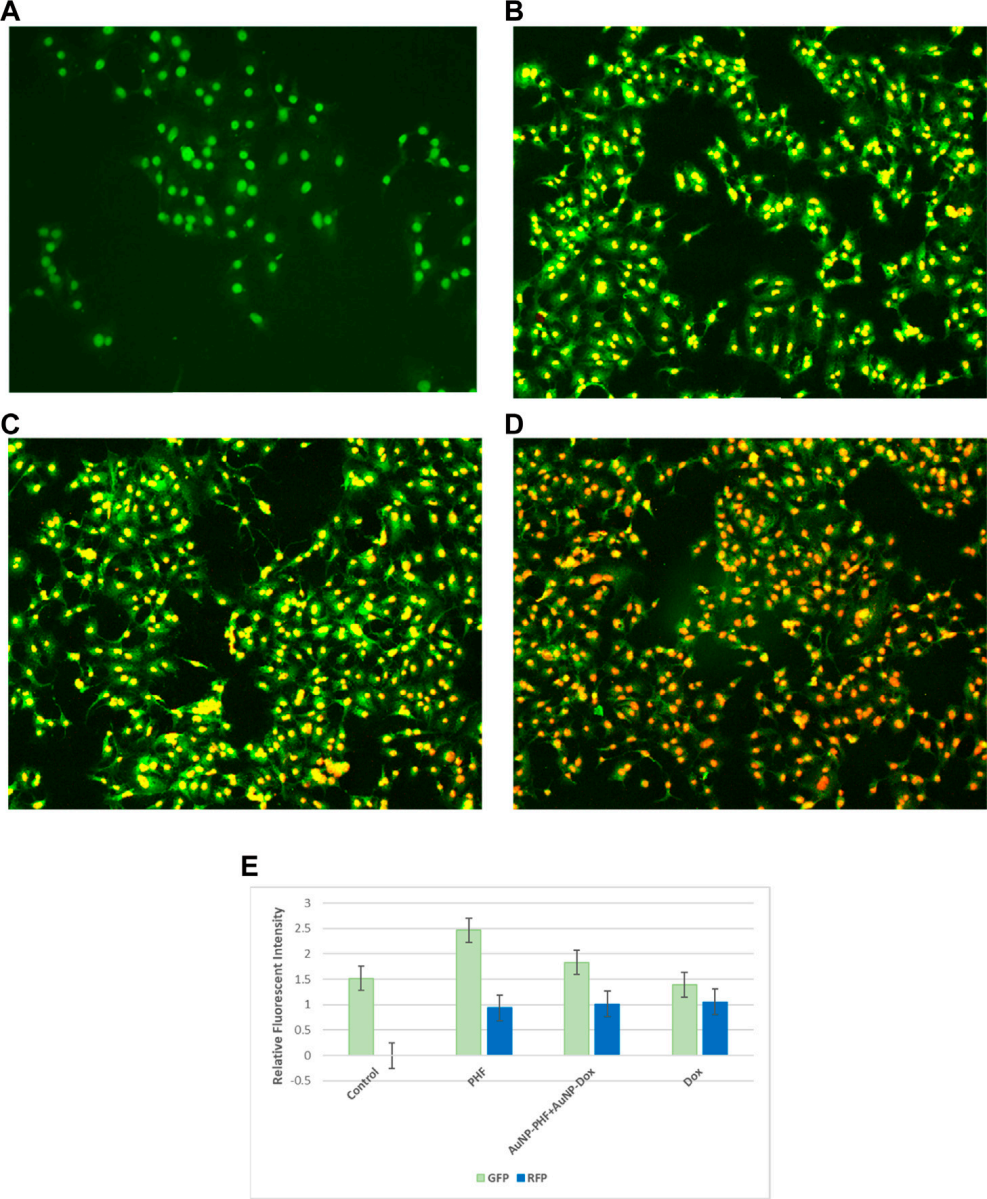
Representative pictures of apoptotic bodies generated in PacR/MCF-7 cell lines. Briefly, the cells were exposed to exposed to PHF (10 **(B)**), AuNP-PHF in combination with AuNP-Dox (10 **(C)**), and Dox (10 **(D)**) for 24 h. Untreated cells were chosen as control (10 **(A)**). **(E)** is the graphical representation of the fluorescent intensity of AO and EtBr stains. After incubation, the cells were stained with cytoplasmic stain acridine orange followed by counterstaining with nuclear stain ethidium bromide. The stained cells were visualized under EVOS FLc inverted fluorescent microscope and the images were recorded at ×10 magnification. Fluorescent images reveal that PHF and AuNP-PHF in combination with AuNP-Dox treated PacR/MCF-7 cells induced early apoptosis, while Dox treated PacR/MCF-7 cells induced late apoptosis. (*p* < 0.05)

To visualise apoptotic cells, both treated and untreated PacR/MCF-7 cells were stained with dual dyes to stain both cytoplasm and nuclei. Acridine orange was used to stain the cytoplasm, while ethidium bromide was used to stain the nuclei. The underlying principle of the staining is that viable cells comprise double-stranded DNA and emit green fluorescence. In contrast, dead cells have single-stranded DNA and emit yellow (early apoptosis) to red (late apoptosis) fluorescence. PacR/MCF-7 cells treated with IC50 PHF and AuNP-PHF in combination with AuNP-dox showed crescent shaped yellow-green AO fluorescent indicating early apoptosis. While PacR/MCF-7 cells treated with IC50 dox resulted in late-apoptosis characterized by concentrated and asymmetrically localized orange nuclei. Overall, our results suggest that PacR/MCF-7 treated with Dox induced late apoptosis, while the other combination of drugs induced early apoptosis (Figure 10). To quantify apoptotic bodies generated as a result of AO/EtBr staining, the fluorescence intensity was quantified using ImageJ software and the results were represented in bar diagram as shown in the panel (e) of Figure 10.

### 3.13 Cellular ROS assay

**FIGURE 11:**
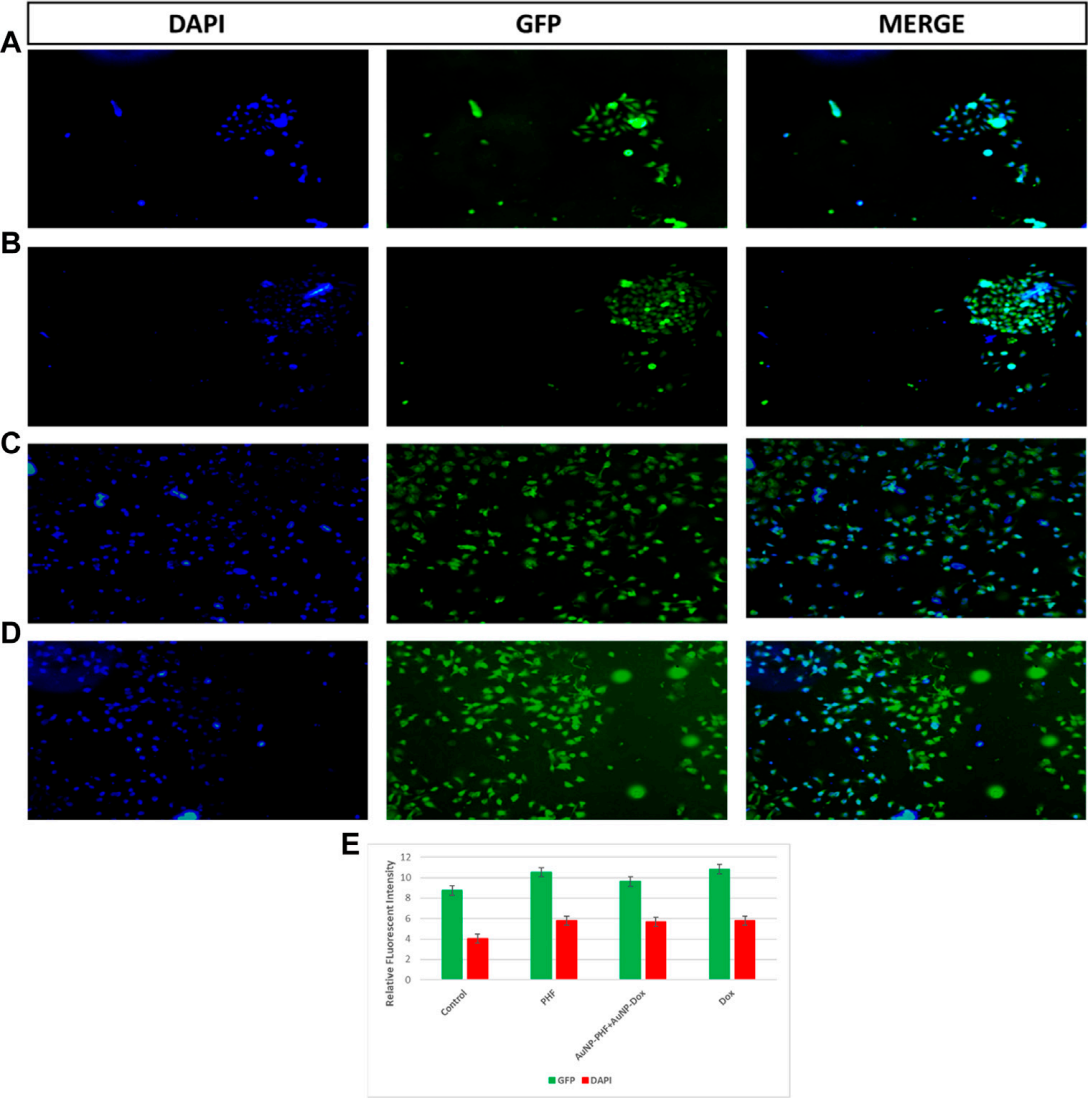
Representative pictures of cellular ROS generated in PacR/MCF-7 cell lines. Briefly, the cells were exposed to exposed to PHF (11 **(B)**), AuNP-PHF in combination with AuNP-Dox (11 **(C)**), and Dox (11 **(D)**) for 24 h. Untreated cells were chosen as control (11 **(A)**). **(E)** is the graphical representation of the fluorescent intensity of DCF-DA and Hoechst 33342 stains. After incubation, the cells were stained with DCF-DA that specifically emits fluorescence in presence of cellular ROS followed by counterstaining with nuclear stain Hoechst 33342. The stained cells were visualized under EVOS FLc inverted fluorescent microscope and the images were recorded at ×10 magnification. Fluorescent images reveal that all the treated cells showed elevated cellular ROS with pyknotic nuclei. While control cells showed large nuclei with faint green fluorescence (*p* < 0.05).

Targeting the mitochondria is the characteristic feature to sensitize tumor cells to cellular death pathways, to determine whether the anti-proliferative effect of the chosen drugs was mediated by the generation of cellular ROS, the ROS levels in PacR/MCF-7 was assessed by DCF-DA fluorescent staining. To visualize the generation of cellular ROS, both drug-treated and untreated PacR/MCF-7 cell lines were stained with DCF-DA, that selectively stains cellular ROS. Detection of cellular ROS by DCF-DA depends on diffusion. Once, DCF-DA enters into the cell, it undergoes deacetylation to form a non-fluorescent compound which is mediated by cellular esterase. This compound, when reacts with ROS forms highly fluorescent molecule named as 2′,7′ dichlorofluorescein (DCF). The intensity of fluorescence emitted by the cells depend on the levels of cellular ROS generated. The stained cells were visualised under EVOS FLc inverted fluorescent microscope and the pictures were recorded at ×10 magnification. As shown in Figure 11, PacR/MCF-7 cells treated with the desired combination of drugs showed pyknotic nuclei with green cytoplasm indicating ROS suggesting that both the drugs individually and in combination induced ROS production. To quantify cellular ROS generated as a result of DCF-DA staining, the fluorescence intensity was quantified using ImageJ software and the results were represented in bar diagram as shown in the panel (e) of Figure 11.

## 4 Discussion

Poor solubility is a major concern for the bioavailability of natural bioactive compounds such as phytosterols, flavonoids, etc (Lee et al., 2018; Pandrangi et al., 2022b). Various types of drug delivery approaches have been established to overcome this problem. Among them, a nanoparticle-based drug delivery system is more efficient in delivering hydrophobic drugs into the cells without degrading them in circulation. Due to their advantages with respect to the surface characterization that permits easy functionalization with biological as well as synthetic compounds accompanied with low toxicity, gold nanoparticles have gained more attention as drug delivery vehicles (Amreddy et al., 2018). Targeted drug delivery facilitates controlled release of the drug in precise amounts at the targeted sites. This approach not only enhances the efficacy of the drug but also reduces the overall dose, which ultimately leading to unwarranted side effects caused by the anti-cancer agents (Yang et al., 2021). On the other hand, Soumen Das et al. (2012) have demonstrated that consumption of swarna bhasma which contains gold nanoparticles showed better prognosis and survival outcome in rectal cancer group.

Our results from the characterization studies reveal that PHF and dox loaded into AuNPs showed higher stability with a zeta potential of −0.6 mV and 0.2 mV and with a particle size of 35.1 nm and 1.9 nm, respectively. According to the literature, AuNPs loaded with various drugs with zeta potential ranging from −30 to + 30 mV show high stability. Additionally, the literature suggests that the particle size of AuNPs less than 50 nm could easily penetrate the nucleus and shows its cytotoxicity on the DNA. Cell cycle analysis demonstrated that the AuNP-PHF and AuNP-Dox potentially induced G0/G1 arrest, which signifies DNA fragmentation. All these results demonstrate that the loaded drugs potentially penetrated the nucleus and induced DNA damage. To further elucidate the mechanism through which the selected drugs have induced cell death, gene expression analysis was carried out to evaluate the expression of ferritinophagy genes.

Ferritinophagy is a novel mechanism that depends on the autophagic degradation of ferritin. Ferritin is an iron-storage protein found in large quantities, particularly in cancer and cancer stem cells (CSCs), to sustain the vital tumor microenvironment (Ryu et al., 2018). Degrading ferritin reserves paved a novel pathway to induce cell death in cancer and CSCs by activating lipoxygenase and Fenton reactions. Fenton reactions play a vital role in inducing tumor cell death by elevating ROS, while lipoxygenase catalyzes the generation of lipid ROS, which is the hallmark of ferroptosis (Yang et al., 2014). Ferritinophagy and ferroptosis are similar cell death pathways, and the only difference is that in ferritinophagy, ferritin degradation is mediated by the activation of autophagy.

Tumor relapse/recurrence is one of the major reasons for increasing cancer incidence leading to poor survival outcomes. The major factor responsible for tumor relapse/recurrence is the presence of CSCs (Resende and Ulrich, 2013; Pandrangi et al., 2014b; Malla et al., 2018). CSCs are the major drivers for enhanced proliferation, differentiation, EMT transition, acquired resistance to conventional therapies and apoptosis (Aktas et al., 2009). Hence, in the present study we focused on generation paclitaxel-resistant breast cancer stem cells which are genotypically identical to CSCs. As expected AuNP-PHF in combination with low concentration of AuNP-Dox re-sensitized PacR/MCF-7CSCs to cell death and induced ferritinophagy.

The main purpose of the present study is to achieve greater solubility and reduce the concentration of dox. Dox is one of the conventional anti-neoplastic drugs used for various malignancies (Micallef and Baron, 2020). However, adverse side effects of dox are the major issue for the increased mortality rate caused due to secondary infections. Cardiotoxicity is one of the major concerns that is affecting overall patient survival (Thorn et al., 2011; Zhao and Zhang, 2017; Chikati et al., 2018; Karabicici et al., 2021). In our previous studies, we demonstrated that aqueous Theobroma extract, when used in combination with low levels of dox (IC25), induced ferroptosis in carboplatin-resistant cervical cancer stem cells (Chittineedi et al., 2022b). Hence, in the present study, we hypothesized that loading the drugs (PHF and dox) into AuNPs might enhance the solubility of the drug and delivers the drug into the target site there by reducing the concentration of drug that is to be used accompanied with enhanced drug efficacy.

## 5 Conclusion and future prospects

Based on our results we conclude that loading the anti-cancer drugs into AuNPs, enhanced the stability of these drugs accompanied with reduced IC50. Additionally, combination therapy of PHF with dox reduced the IC50 of dox drastically leading to minimal side-effects. However, further *in vivo* studies are needed to be done to check the potential of these drugs to induce ferritinophagy without any side effects in animal models.

## Data Availability

The original contributions presented in the study are included in the article/supplementary materials, further inquiries can be directed to the corresponding authors.

## References

[B1] Aktas B. Tewes M. Fehm T. Hauch S. Kimmig R. Kasimir-Bauer S. (2009). Stem cell and epithelial-mesenchymal transition markers are frequently overexpressed in circulating tumor cells of metastatic breast cancer patients. Breast Cancer Res. 11 (4), R46. 10.1186/bcr2333 19589136 PMC2750105

[B2] Amreddy N. Babu A. Muralidharan R. Panneerselvam J. Srivastava A. Ahmed R. (2018). Recent advances in nanoparticle-based cancer drug and gene delivery. Adv. Cancer Res. 137, 115–170. 10.1016/bs.acr.2017.11.003 29405974 PMC6550462

[B3] Atanasov A. G. Waltenberger B. Pferschy-Wenzig E. M. Linder T. Wawrosch C. Uhrin P. (2015). Discovery and resupply of pharmacologically active plant-derived natural products: A review. Biotechnol. Adv. 33 (8), 1582–1614. 10.1016/j.biotechadv.2015.08.001 26281720 PMC4748402

[B4] Basuli D. Tesfay L. Deng Z. Paul B. Yamamoto Y. Ning G. (2017). Iron addiction: A novel therapeutic target in ovarian cancer. Oncogene 36 (29), 4089–4099. 10.1038/onc.2017.11 28319068 PMC5540148

[B5] Biazar E. Majdi A. Zafari M. Avar M. Aminifard S. Zaeifi D. (2011). Nanotoxicology and nanoparticle safety in biomedical designs. Int. J. Nanomedicine 6, 1117. 10.2147/IJN.S16603 21698080 PMC3118686

[B6] Chanvorachote P. Luanpitpong X. S. (2022). Cell and Molecular Processes in Cancer Metastasis Iron induces cancer stem cells and aggressive phenotypes in human lung cancer cells. Am. J. Physiol. Cell Physiol. 310, C728–C739. 10.1152/ajpcell.00322.2015 26911281

[B7] Chen Y. Fan Z. Yang Y. Gu C. (2019). Iron metabolism and its contribution to cancer (Review). Int. J. Oncol. 54 (4), 1143–1154. 10.3892/ijo.2019.4720 30968149

[B8] Chikati R. Pandrangi L. S. Gundampati R. Vemuri S. H. Lakhanpal M. Singh S. S. (2018). Molecular studies on evaluation of phytol as cytoskeleton targeting element in cancer. Int. J. Sci. Eng. Res. 9 (10), 1978–1992.

[B9] Chittineedi P. Latha Pandrangi S. Mohiddin G. J. Neira Mosquera J. A. Sánchez Llaguno S. N. (2022). Concomitant therapy of aq. Theobroma extract and doxorubicin reduces stemness and induces ferroptosis in therapeutic resistant cervical cancer cells. J. Carcinog. Mutagen. 10.35248/2157-2518.22.S32:001

[B10] Chittineedi P. Pandrangi S. L. Bellala R. S. Naynee S. Llaguno S. Neira Mosquera J. A. (2022). Analyzing the drivers of cancer relapse: Hypocalcemia and iron absorption in hormone-dependent female cancers. Am. J. Transl. Res. 14 (9), 6563–6573. 36247282 PMC9556499

[B11] Das S. Das M. Paul R. (2012). Swarna bhasma in cancer: A prospective clinical study. AYU (An International Quarterly Journal of Research in Ayurveda) 33 (3), 365–367. 10.4103/0974-8520.108823 PMC3665104 23723642

[B12] Gao M. Monian P. Pan Q. Zhang W. Xiang J. Jiang X. (2016). Ferroptosis is an autophagic cell death process. Cell Res 26 (9), 1021–1032. 10.1038/cr.2016.95 27514700 PMC5034113

[B13] Hermann P. C. Bhaskar S. Cioffi M. Heeschen C. (2010). Cancer stem cells in solid tumors. Semin Cancer Biol 20 (2), 77–84. 10.1016/j.semcancer.2010.03.004 20371287

[B14] Karabicici M. Alptekin S. Fırtına Karagonlar Z. Erdal E. (2021). Doxorubicin-induced senescence promotes stemness and tumorigenicity in EpCAM−/CD133− nonstem cell population in hepatocellular carcinoma cell line, HuH-7. Mol Oncol 15 (8), 2185–2202. 10.1002/1878-0261.12916 33524223 PMC8334288

[B15] Latha Pandrangi S. Shree Chalumuri S. Chittineedi P. Garimella S. V. leader G. (2022). Therapeutic potential of nyctanthes arbor-tristis on cancer and various diseases. Cell Biology 26, 1690–1701.

[B16] Lee B. Moon K. M. Kim C. Y. (2018). Tight junction in the intestinal epithelium: Its association with diseases and regulation by phytochemicals. J Immunol Res 2018, 2645465. 10.1155/2018/2645465 30648119 PMC6311762

[B17] Li Y. Wang Z. Ajani J. A. Song S. (2021). Drug resistance and Cancer stem cells 5, 1–11. 10.1186/s12964-020-00627-5 PMC7885480 33588867

[B18] Malla R. R. Pandrangi S. Kumari S. Gavara M. M. Badana A. K. (2018). Exosomal tetraspanins as regulators of cancer progression and metastasis and novel diagnostic markers. Asia Pac J Clin Oncol 14 (6), 383–391. 10.1111/ajco.12869 29575602

[B19] Micallef I. Baron B. (2020). Doxorubicin: An overview of the anti-cancer and chemoresistance mechanisms.

[B20] Pandrangi S. L. Chalumuri S. S. Garimella S. (2022). Emerging therapeutic efficacy of alkaloids as anticancer agents. Ann Rom Soc Cell Biol 26 (01), 64–74.

[B21] Pandrangi S. L. Chikati R. Chauhan P. S. Kumar C. S. Banarji A. Saxena S. (2014). Effects of ellipticine on ALDH1A1-expressing breast cancer stem cells-An *in vitro* and *in silico* study. Tumor Biology 35 (1), 723–737. 10.1007/s13277-013-1099-y 23982874

[B22] Pandrangi S. L. Chittineedi P. Chalumuri S. S. Meena A. S. Mosquera J. A. N. Llaguno S. N. S. (2022). Role of intracellular iron in switching apoptosis to ferroptosis to target therapy-resistant cancer stem cells. Molecules 27, 3011. 10.3390/molecules27093011 35566360 PMC9100132

[B23] Pandrangi S. L. Raju Bagadi S. A. Sinha N. K. Kumar M. Dada R. Lakhanpal M. (2014). Establishment and characterization of two primary breast cancer cell lines from young Indian breast cancer patients: Mutation analysis. Cancer Cell Int 14 (1), 14–20. 10.1186/1475-2867-14-14 24502646 PMC4016554

[B24] Rajesh S. Lillard J. W. (2000). Nanoparticle-based targeted drug delivery. Exp Mol Pathol 86 (3), 215–223. 10.1016/j.yexmp.2008.12.004 PMC3249419 19186176

[B25] Rambatla P. K. Pandrangi S. L. Rentala S. Sireesha V. (2021). A study on the expression of CCL5, CXCR4 and angiogenic factors by prostate cancer stem cells. 25, 1020–1028.

[B26] Recalcati S. Gammella E. Cairo G. (2019). Dysregulation of iron metabolism in cancer stem cells. Free Radic Biol Med 133, 216–220. 10.1016/j.freeradbiomed.2018.07.015 30040994

[B27] Resende R. R. Ulrich H. (2013). Trends in stem cell proliferation and cancer research. Trends in Stem Cell Proliferation and Cancer Research, 1–661.

[B28] Ryu M. S. Duck K. A. Philpott C. C. (2018). Ferritin iron regulators, PCBP1 and NCOA4, respond to cellular iron status in developing red cells. Blood Cells Mol Dis 69, 75–81. 10.1016/j.bcmd.2017.09.009 29032941 PMC5783766

[B29] Thorn C. F. Oshiro C. Marsh S. Hernandez-Boussard T. McLeod H. Klein T. E. (2011). Doxorubicin pathways: Pharmacodynamics and adverse effects. Pharmacogenet Genomics 21 (7), 440–446. 10.1097/FPC.0b013e32833ffb56 21048526 PMC3116111

[B30] Wang Y. Yu L. Ding J. Chen Y. (2019). Iron metabolism in cancer. Int J Mol Sci 20 (1), 95–22. 10.3390/ijms20010095 PMC6337236 30591630

[B31] Wicki A. Witzigmann D. Balasubramanian V. Huwyler J. (2015). Nanomedicine in cancer therapy: Challenges, opportunities, and clinical applications. Journal of Controlled Release 200, 138–157. 10.1016/j.jconrel.2014.12.030 25545217

[B32] Yang J. Jia C. Yang J. (2021). Designing nanoparticle-based drug delivery systems for precision medicine. Int J Med Sci 2021 (13), 2943–2949. 10.7150/ijms.60874 PMC8241788 34220321

[B33] Yang W. S. Sriramaratnam R. Welsch M. E. Shimada K. Skouta R. Viswanathan V. S. (2014). Regulation of ferroptotic cancer cell death by GPX4. Cell 156 (1–2), 317–331. 10.1016/j.cell.2013.12.010 24439385 PMC4076414

[B34] Zhao L. Zhang B. (2017). Doxorubicin induces cardiotoxicity through upregulation of death receptors mediated apoptosis in cardiomyocytes OPEN. Germany: Nature Publishing Group. 10.1038/srep44735 PMC5353581 28300219

[B35] Zheng Y. Li Z. Chen H. Gao Y. (2020). Nanoparticle-based drug delivery systems for controllable photodynamic cancer therapy. European Journal of Pharmaceutical Sciences 144, 105213. 10.1016/j.ejps.2020.105213 31926941

